# Value and specificity of rare diseases business model-is the pursuit of this societal priority sustainable?

**DOI:** 10.1186/1750-1172-7-S2-A23

**Published:** 2012-11-22

**Authors:** M Dunoyer, P Rollet

**Affiliations:** 1 GlaxoSmithKline Rare Diseases, London, UK

## 

With less than 90 diseases covered by approved treatments in Europe for approximately 7,000 rare diseases described, patients with rare diseases are underserved.

Although western societies attribute value to equality of access irrespective of an individual’s ability to pay, affordability of OMPs (Orphan Medicinal Products) has been the subject of a health policy debate[1]. The debate is exacerbated by perceived high acquisition costs for OMPs whereas their budget impact is low [2,3]. It is essential to address misconceptions about the rare diseases model to understand how to make it sustainable.

OMPs development drives value to patients by providing access to innovative treatments in untapped diseases. In France, the relative share of innovative OMPs reimbursed between 2001 & 2009 was almost twice as high as common diseases drugs and most of them targeting diseases with no approved treatment [3].

OMPs budget impact is likely to remain low [2]. A few drugs with low price ranges represent largest budget impact of OMPs [4]. Population size to be treated rather than individual drug prices influences budget impact in rare diseases.

The development of OMPs is a risky undertaking for companies that invest in this field. Due to the small patient population, development timelines on OMPs are similar to that of other drugs [5]. The conduct of clinical trials in rare diseases is often more expensive on a per patient basis. R&D investment must therefore be recovered with lower volume of sales, leading to higher prices for OMPs than other medicines.

Scientific advances offer new perspectives by making treatable and curable new important diseases. Policy makers must reward innovation based upon unmet need and patient outcome. Technology platforms, franchises and global reach are three potential levers at company level to sustain profitable development of OMPs. New and innovative pricing models based on rational value assessment and realized by contractual agreements could address the issue of affordability while taking into account country differences.

While there are strategic choices that manufacturers can make to overcome barriers to contribute to public health goals, there are many other factors that inhibit effective or optimal access to new treatments that are beyond industry’s control or influence.

Social, ethical, political considerations must guide improved access so that all patients can access OMPs in an equitable manner. Political will is needed to allocate the necessary funding within health budgets and support a solid rare diseases public health policy.
